# Psychometric Properties and Gender Invariance of the Positive Mental Health Scale in Spanish Nurses During the COVID‐19 Pandemic

**DOI:** 10.1002/nop2.70185

**Published:** 2025-04-14

**Authors:** María Auxiliadora Robles‐Bello, David Sánchez‐Teruel, Selma Boufellous, Cristina Lendínez‐Rodríguez

**Affiliations:** ^1^ Psychology Department University of Jaen Jaen Spain; ^2^ Faculty of Psychology University of Granada Granada Spain

**Keywords:** critical situations, gender differences, healthcare professionals, psychological variables, psychometrics characteristics

## Abstract

**Aim:**

Positive mental health (PMH) can be considered a key aspect of mental health in the face of potentially stressful healthcare situations such as the COVID‐19 pandemic. The aim of this study was to analyse the psychometric properties of the Spanish version of the Positive Mental Health Scale (PMS) in Spanish nurses during the COVID‐19 pandemic.

**Design:**

Descriptive analysis, confirmatory factor analysis, gender invariance analysis and convergent and divergent analyses were performed, and reliability indices were calculated.

**Methods:**

A total of 661 nurses (425 women and 236 men) participated in the study. They completed various questionnaires during August–October 2021.

**Results:**

Factor analysis demonstrated a unidimensional structure with very good indices of model fit, high positive convergent validity, especially with social support, self‐efficacy and resilience to suicide attempts, and high divergent validity with anxiety and, to a lesser extent, with depression. There was also strong invariance between genders and high reliability indices. In conclusion, the data show that the PMS has adequate validity and reliability in nurses. Furthermore, this study allows us to confirm gender invariance, which has not been examined in other studies. The data show that PMS is a suitable measure for assessing the mental health of healthcare professionals exposed to high‐stress situations.

**Patient or Public Contribution:**

No patient or public contributions.

## 1 Introduction

The COVID‐19 pandemic had a great impact on the world's population, and especially on some specific groups. The continued and exponential increase in infections overwhelmed health systems and forced healthcare professionals to deal with a critical, highly stressful situation that had a great socioemotional impact and triggered the deterioration of their mental health (Braquehais et al. 2020; Dosil et al. 2021).

## 2 Background

Among healthcare workers, nurses deserve special attention because they were on the front line, receiving and providing direct care to infected patients and preventing the spread of the disease. During the pandemic, these professionals were exposed to a significant increase in workload, a lack of personal protective equipment, and being shifted from their usual work units to units caring for suspected or infected COVID‐19 patients, resulting in feelings of a lack of support, an enormous psychological workload (Lai et al. 2020), and acute stress (Shechter et al. 2020). In addition, nurses were also affected by the pandemic in their personal lives (Hickling and Barnett 2022). They were directly exposed to a disease that caused severe symptoms, had no specific treatment, and was sometimes fatal, and they feared becoming infected or spreading the virus to family and friends. Studies in China found that nurses suffered more psychological distress than other health professionals (Lai et al. 2020; Sarhani‐Robles et al. 2024). Shechter et al. (2020) found a higher percentage of psychological distress in nurses than in physicians. The experience of all these highly stressful situations resulted in psychological distress (Al Maqbali et al. 2021), most frequently anxiety and depression (Wu et al. 2020).

The importance of mental health in nursing is a critical issue that affects both health professionals and patients (Jilili et al. 2024). Nurses are constantly exposed to complex and emotionally draining situations. Whether caring for terminally ill patients, witnessing the difficulties of those with chronic illnesses, or supporting people who have suffered severe trauma, it is vital to recognise the impact these experiences can have on their emotional well‐being (Jilili et al. 2024; Piras et al. 2024). Maintaining good mental health is essential to providing quality care and to looking after one's own long‐term well‐being. Being tired, anxious or stressed not only affects your physical health, but also your ability to make clear decisions and carry out your work effectively. In this context, Velten et al. (2022) considers that nurses play a crucial role in the delivery of health services, as they dedicate their lives to providing care and meeting basic daily needs at all stages of a population's development. Therefore, it is of paramount importance to know the mental health status of nurses from the perspective of optimising general well‐being, that is, positive mental health.

In this regard, it would be interesting to assess protective factors that promote positive mental health (hereafter PMH) in the face of potentially stressful situations. The term positive mental health was first coined by Jahoda (1958) to denote a person's positive feelings and psychological, emotional and social well‐being (Keyes et al. 2002). In a positive social context, factors associated with people being healthy include: the ability to manage time, having meaningful social relationships, working effectively with others, having high self‐esteem and being proactive. PMH has also been related to the ability to think and communicate with people and to being able to recognise, understand and interpret different contexts, adapt to them and change them when necessary (Roldán‐Merino et al. 2017). Positive mental health can be considered an aspect related to mental health, as it is associated with an individual's overall health status and could be explored or enhanced. In nursing students, PMH status has been shown to be important in developing personal and professional competencies (Nami et al. 2014). However, there are few instruments measuring PMH, and even fewer for measuring it in nurse health professionals. One such tool is the PMH‐Scale, which is based on the two components: the hedonic tradition, which refers to positive affect and high levels of life satisfaction, and the eudaimonic tradition, which refers to a person's adequate functioning in everyday life (Deci and Ryan 2008).

The PMH scale is a unidimensional scale composed of 9 Likert‐type items (Lukat et al. 2016). This scale was adapted in several studies using a population of students from Germany, a population of psychiatric patients from Germany who received cognitive behavioural therapy, and a general sample of participants (also from Germany) with and without diagnoses of mental disorders or in remission (Lukat et al. 2016). That study found optimal correlations with variables related to life satisfaction.

In the same vein, a study was designed to investigate the psychometric properties of the PMH in Turkish university students using a cross‐sectional correlational survey model. The results showed significant positive correlations between the PMH and optimism, happiness and general self‐efficacy; in contrast, there were significant negative correlations with depression, anxiety and stress. These results indicate that it has good convergent validity (Çeçen and Vatandaşlar 2021). This has also been demonstrated in recent studies in different cultural contexts (Brailovskaia and Margraf 2020) and even when suicidal ideation is assessed (Brailovskaia et al. 2020).

This scale was validated in various populations during the COVID‐19 pandemic (Brailovskaia and Margraf 2020), including the Spanish population, where it demonstrated high levels of reliability and validity (Boufellous et al. 2023). That particular study found high convergent validity with the variable optimism and resistance to suicide, and high negative convergent validity with anxiety. However, to our knowledge, the scale has not been validated with a specific population, such as nurses.

Therefore, the main objective of this study was to evaluate and validate the psychometric properties of the Positive Mental Health scale in Spanish nurses, in the context of the COVID‐19 pandemic. More specifically, the aim was to analyse the structure and internal consistency of the scale and its gender invariance in this population.

## 3 Method

### 3.1 Study Design

The study design was descriptive, instrumental and cross‐sectional to assess the reliability, validity and invariance of PMH in nurses.

### 3.2 Participants

From an initial sample of 920 participants, 259 were discarded as they did not meet any of the inclusion criteria. These criteria were: (1) More than 1 year working as a nurse; (2) Having been exposed to stressful situations during the onset of the COVID‐19 pandemic; (3) Resident in Spain; (4) Reading and signing the informed consent form. The final sample comprised 661 participants, 425 women and 236 men between 27 and 62 years old (*M* = 32.1; SD = 4.2). Table 1 presents the sociodemographic data.

**TABLE 1:** Description of sociodemographic data.

Variable	*N* (%)	Contrast	df	Phi
Gender		5.63^ns^	1	0.42
Female	425 (50.30)			
Male	236 (49.70)			
Age		2.11^ns^	2	0.62
27–39	199 (23.55)			
40–52	268 (31.71)			
53 or more	194 (22.96)			
Marital status		1.27**	5	0.73
Single	97 (14.67)			
Domestic partner	86 (13.01)			
Married	254 (38.43)			
Separated/divorced	195 (29.50)			
Widowed	23 (3.48)			
Others	6 (0.91%)			
Number of children		1.02**	3	0.84
0	138 (20.88)			
1	376 (56.88)			
2	124 (18.76)			
3 or more	23 (3.48)			
Total	661			

*Note:* Contrast, Chi‐squared/Student *t*; *p* < 0.05.

Abbreviations: d.f., degrees of freedom; ns, not significant; Phi, effect size.

** Refers to the significance level < 0.01 (bilateral).

### 3.3 Measures

*Sociodemographic data sheet*. A data sheet was prepared for this study to capture information on gender, age, marital status and number of children.

*Positive Mental Health (PMH)* by Lukat et al. (2016). This measures positive psychosocial well‐being, where the higher the score the more positive the mental health. This unidimensional scale is composed of 9 items each with a 4‐point Likert‐type response (e.g., ‘I enjoy my life’; 0 = disagree to 3 = agree). It was adapted to the general Spanish population (Boufellous et al. 2023) (see Appendix 1) using a sample of 845 people (425%–50.30% women) aged between 14 and 70 years old (*M* = 32.1; SD = 4.2). Descriptive analyses of the items were carried out, as well as confirmatory factor analysis and convergent validity with protective factors and with risk factors. That study concluded that the PMH could be an appropriate measure for assessing positive mental health in health professionals. It shows high levels of reliability in the original version among university students and the general population in Germany (alpha = 0.92, alpha = 0.93).

*Hospital, Anxiety and Depression (HAD‐14)* in the version by Snaith and Zigmond (1986), translated into Spanish by Herrero et al. (2003). This is a 14‐item scale designed to assess anxiety and depression in nonpsychiatric hospital outpatient services. It is a state measure containing two scales, one for anxiety (A) and one for depression (D). It is a useful instrument that has been validated in Spain and is of special interest and utility in the context of Primary Care. It has a 7‐item A subscale and a 7‐item D subscale using a 4‐point Likert‐type format, giving maximum scores of 21 for each subscale. The questionnaire assesses symptoms during the previous week. The scale has good internal consistency of 0.90 according to Cronbach's alpha for the full scale, 0.84 for the depression subscale and 0.85 for the anxiety subscale. In the present study, the alpha for the total instrument was 0.89 and the reliability was also adequate for the subscales (*α*A = 0.89; *α*D = 0.81).

The *General Self‐Efficacy Scale‐GSE* (Schwarzer and Jerusalem 1995) was translated into Spanish as *Escala de Autoeficacia General* by Sanjuán et al. (2000). This scale measures general self‐efficacy, that is, the belief that one's own actions are responsible for successful outcomes. It has 10 items with responses from 1 (completely false) to 4 (completely true). Scores range from 10 to 40 points, where the higher the score, the higher the perceived overall self‐efficacy. The internal consistency of the Spanish version was 0.84, and, in our study, Cronbach's alpha was 0.96.

The *Multidimensional Scale of Perceived Social Support* (MSPSS) by Zimet et al. (1988) was adapted to Spanish by Landeta and Calvete (2002). This 12‐item instrument with 7 response alternatives (where 1 is ‘strongly disagree’ and 7 is ‘strongly agree’) measures the subject's perceived levels of social support through three subdimensions: family, friends and partner. Having high scores on each of the subscales indicates higher levels of perceived social support, and the total of the three scales produces an overall scale of satisfaction with perceived social support. The reliability of the original study in samples of college students was 0.85 (Cronbach's alpha), and in subsequent studies also with college students by McDonald (1999) it was 0.93 (Osman et al. 2014). In our study, alpha was 0.98.

The *Scale of Resilience to Suicide Attempts* (SRSA‐18) by Sánchez‐Teruel et al. (2021). This instrument was constructed to assess resilience in a Spanish clinical population with previous suicide attempts and validated to verify its efficacy in predicting future suicide attempts within 6 months. The scale has 18 items in three subdimensions (internal and external protection and emotional stability). It provides a short, rapid evaluation through protective factors instead of risk factors of the level of resilience to suicide attempts and can help prevent more lethal future suicide attempts. It is able to predict future suicide attempts at 6 months in people who have made previous attempts. Its level of internal consistency is adequate (*α* = 0.88; *ω* = 0.89) and it has concurrent validity with scales of general resilience (CD‐RISC and RS‐14) and resilience in suicidal ideation (SRI‐25) (Sánchez‐Teruel et al. 2021). In our study, Cronbach's alpha was 0.79.

### 3.4 Data Collection

The procedure was carried out in several steps. Firstly, questionnaires were distributed through various social networks, indicating that the study was intended for nurses, but the google form was also distributed by mail to health centres and public and private hospitals. Data collection took place during August–October 2021.

### 3.5 Ethical Considerations

This study was approved by the research bioethics committee at the university of one of the authors (reference: DEC.20/9.TFM; JUL.22/5.LINE). The ethical guidelines of the General Council of the Official College of Psychologists (2023) and the principles of the Declaration of Helsinki (World Medical Association 2013) were followed. Informed consent was obtained from all participants included in the study, without which they were unable to complete the evaluation measures.

### 3.6 Statistical Analysis

First, a multiple imputation method was applied to missing values (< 1.5%) (Lorenzo‐Seva and Van Ginkel 2016) and the Mahalanobis distance was used to assess the existence of outliers (Tabachnick and Fidell 2018). This was followed by a descriptive analysis of the items. Next, confirmatory factor analysis (CFA) was carried out using SPSS with AMOS 23 (I.B.M. Corporation 2013). The CFA used a polychoric correlation matrix with generalised least squares (GLS). The fit indices were the *χ* ^2^/df ratio, the root mean square error of approximation (RMSEA), the goodness‐of‐fit index (GFI), the comparative fit index (CFI) and the Tucker–Lewis index (TLI). Goodness‐of‐fit is considered satisfactory when TLI and CFI ≥ 0.95, the goodness‐of‐fit index (GFI) approaches 0.90, and the RMSEA approaches 0.05 (Kline 2016).

In addition, it was analysed whether there were differences in the invariance of the measure by gender using multigroup CFA with AMOS. Two nested models were defined for gender. The Satorra–Bentler scale (*χ* ^2^) and its *p* values were used for measurement invariance, together with the RMSEA with a 90% CI and the ΔCFI, as an index of incremental fit (Hooper et al. 2008). Measurement invariance exists when *p* > 0.05 for Δ*χ* ^2^ (considering sample size bias); RMSEA values ≤ 0.05 and the value of ΔCFI for the compared models is < 0.01 (Byrne 2016). A configurational invariance analysis (base model) was performed with factor means set to zero, and scalar invariance was analysed to assess whether the differences between the groups indicated by the items were the same for all items (Van de Schoot et al. 2012). Finally, external evidence of scale validity (criterion validity) was obtained through correlation with measures that are positively and negatively related to PMH. The level of statistical significance required in all tests was a minimum of *p* < 0.05.

## 4 Results

### 4.1 Descriptive Item Analysis

Missing values represented < 1.5% of the total sample, and the multiple imputation method was applied. No outliers were found using the Mahalanobis distance (largest *χ* ^2^ = 2.34; df = 4; *p* > 0.01). The data provided by item analysis and internal consistency showed variability in skewness and kurtosis in this sample (Table 2), indicative of moderate univariate normality. All items correlated well with the total scale (equal to or greater than 0.50), and there was no improvement in the overall reliability of the scale if any of the items were removed.

**TABLE 2:** Descriptive statistics, skewness and kurtosis indices and item analysis.

Item	*M* (SD)	K‐S	S	K	r item‐total	Alpha if the item is deleted
			SE (−0.01)	SE (2.69)		
ITEM 1	1.99 (1.1)	0.91^ns^	0.21	0.32	0.77	0.71
ITEM 2	2.01 (1.12)	0.81^ns^	0.34	0.41	0.75	0.87
ITEM 3	2.79 (0.97)	0.86*	−0.01	0.08	0.83	0.74
ITEM 4	1.98 (0.96)	0.86^ns^	−0.03	−0.06	0.71	0.78
ITEM 5	1.96 (1.20)	0.80^ns^	0.23	0.39	0.78	0.88
ITEM 6	2.20 (0.96)	0.03**	−0.46	−0.39	0.75	0.83
ITEM 7	2.16 (0.89)	0.89^ns^	−0.49	−0.52	0.71	0.80
ITEM 8	2.24 (0.98)	0.90^ns^	0.53	0.68	0.85	0.76
ITEM 9	1.998 (0.83)	0.86^ns^	−0.31	−0.41	0.78	0.79
Total	14.16 (6.42)	0.92^ns^	0.52	0.60	1	0.89

Abbreviations: K, kurtosis; K‐S, Kolmogorov–Smirnov test; *M*, mean; ns, not significant; S, skewness; SD, standard deviation; SE, standard error of skewness and kurtosis.

* Significance < 0.05 (bilateral).

** Significance < 0.01 (bilateral).

### 4.2 Confirmatory Factor Analysis

**FIGURE 1:**
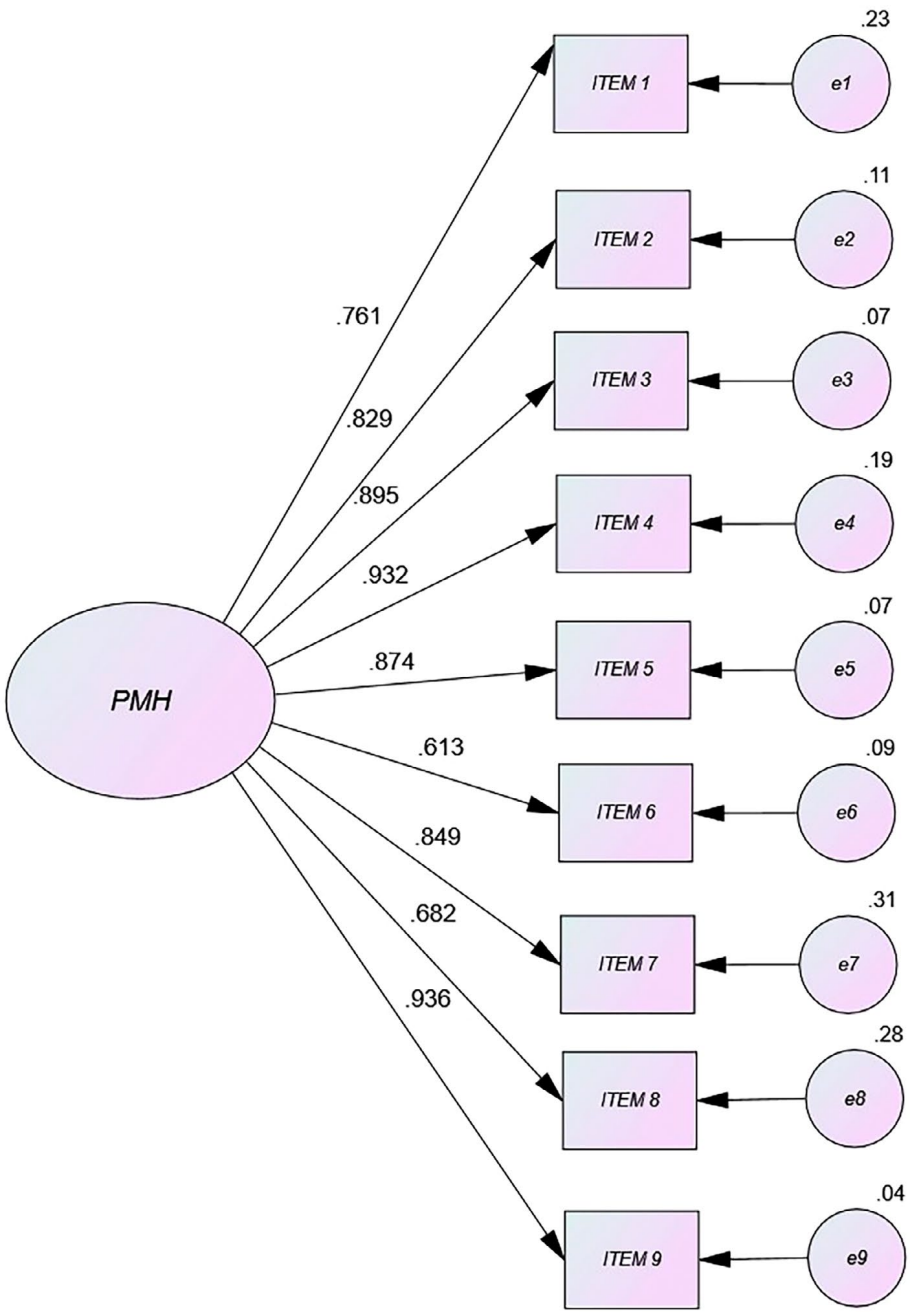
Path diagram of Positive Mental Health‐PMH in nurses.

There was no multivariate normality in the distribution of the items (Mardia = 1526.28) (Mardia 1970). Confirmatory factor analysis gave an adequate and significant *χ* ^2^/df (1.37; *χ* ^2^ = 126.47; degrees of freedom (df) = 92; *p* < 0.01). All other indices were excellent: RMSEA (95% CI) < 0.05 (0.02 [0.01; 0.04]), scores for ΔCFI (0.98) and TLI (0.97) and GFI (0.96), with good agreement between these goodness‐of‐fit indices and a unidimensional structure for the PMH scale. This was confirmed by the results of the path diagram (Figure 1). In short, this measure exhibited a very good fit and excellent indices of acceptability in the sample of Spanish healthcare professionals exposed to stressful situations during the COVID‐19 pandemic.

### 4.3 Gender Invariance Analysis

The invariance analyses (Table 3) show that the CFA models specified for men and women nurses fit the data adequately. The configural invariance test (base model where factor loadings and variances were freely estimated for men and women) and the scalar invariance test (all item intercepts are necessarily equal for all items) also demonstrated good levels of fit. Metric invariance was not assessed as the PMH scale is unidimensional. The results show that there were no significant item differences between men and women, that is, there was no invariance of this measure between genders. Specifically, the increase in *χ* ^2^ from the base model to the scalar invariance model was 1.73, a change that is not statistically significant (Δ*χ* ^2^ = 1.73 (Δdf = 2); *p* > 0.05). The increase in CFI was 0.002, below the criterion of 0.01 (Van de Schoot et al. 2012). Both indices point to full scalar equivalence between men and women, indicating that gender (male and female nurses) does not influence this scale's measurement of positive mental health.

**TABLE 3:** Fit indices for the invariance test by gender.

Variable	*χ* ^2^	df	*p*	RMSEA (95% CI)	CFI	Δ*χ* ^2^	ΔCFI
Men (*n* = 236)	38.09	26	0.03*	0.01 [0.001; 0.012]	0.95		
Women (*n* = 425)	39.27	26	0.01*	0.04 [0.031; 0.052]	0.95		
Configural invariance gender	89.16	45	0.22	0.03 [0.032; 0.043]	0.96		
Scalar invariance gender	87.22	45	0.36	0.03 [0.027; 0.039]	0.97	1.73^ns^ (Δdf = 2)	0.002

Abbreviations: ΔCFI, difference test between Comparative Fit Index; Δ*χ* ^2^, difference test between the configural and scalar invariance models; *χ* ^2^, chi‐squared; CFI, Comparative Fit Index; df, degrees of freedom; ns, not significant; *p*, significance level; RMSEA, root mean square error of approximation.

* *p* < 0.05.

### 4.4 Convergent and Divergent Reliability and Validity

The PMH scale in this sample of nurses exposed to stressful situations due to the COVID‐19 pandemic demonstrated high, significant convergent validity with social support (MSPSS = 0.86; *p* < 0.05), self‐efficacy (GSE = 0.84; *p* < 0.05) and resilience to suicide attempts (SRSA‐18 = 0.79). There was also significant divergent validity with anxiety (HAD‐A = −0.82; *p* < 0.05) and to a lesser extent with depression (HAD‐D = −0.65; *p* < 0.05). In addition, the minimum score was 0, while the maximum was 30, and there was good internal consistency (Cronbach's alpha = 0.96; Omega coefficient = 0.97), showing that the PMH in nurses is a measure with high levels of reliability.

## 5 Discussion

The aim of this study was to analyse the psychometric properties of the PMH scale in Spanish nursing professionals in the context of the COVID‐19 pandemic. Specifically, it was intended to analyse the structure and internal consistency of the scale, as well as its gender invariance in these health professionals.

Initial item analysis of the scale in nurses gave similar results to the original German version in various samples (Lukat et al. 2016) and to adaptations in university students that were subsequently undertaken in other countries and cultures (Brailovskaia et al. 2020; Çeçen and Vatandaşlar 2021). Moreover, our results corroborate the results from applying the same scale to the general Spanish population (Boufellous et al. 2023). The factor structure of the scale was unidimensional and its goodness‐of‐fit indices were excellent in our sample of Spanish healthcare professionals. This suggests that the PMH in nurses has good construct validity, which in turn indicates that the PMH is a concept that can be measured independently of others and therefore exists autonomously with respect to factors that may be related to each other, which is consistent with previous results for this scale (Boufellous et al. 2023; Lukat et al. 2016). It also implies that, if this scale continues to be used to measure PMH in other populations, it may become universal and therefore be taken into account as an important factor in understanding people's mental health.

One important contribution of our study is that it allowed us to examine the gender invariance of the measure in nurses. Overall, the fit indices suggest a good degree of invariance between genders in the model, especially in configurational variance. However, in scalar variance, although the fit indices were quite similar, there was a small difference between *χ* ^2^ and *p*, suggesting that the values were not completely equivalent, but there was a significant degree of invariance between genders in the model. These results are consistent with a previous study looking at Portuguese and Spanish nursing students that found no significant gender differences (Sequeira et al. 2020). Similar results were also found in Turkish and German students (Çeçen and Vatandaşlar 2021; Vatandaşlar et al. 2022), as well as in a cross‐cultural study in the general population in France, Germany, Poland, Russia, Spain, Sweden the United Kingdom and the United States (Velten et al. 2022) that supports the results obtained in this study on gender invariance. In addition, it was found that the overall PMH score was lower in nurses than in the general population. This is consistent with the stressful circumstances these professionals were exposed to during the COVID‐19 pandemic.

In addition, it can also be determined that PMH was strongly correlated with protective factors such as social support and self‐efficacy. It also correlated with resilience, confirming what other studies with university students have suggested about this variable (Bibi et al. 2020; Brailovskaia et al. 2020; Hu et al. 2022), which are in line with those found in the general population by Boufellous et al. (2023) where there was a high correlation with resilience and a moderate correlation with general self‐efficacy and perceived social support. Furthermore, the relationship that was found between social support and PMH corroborates other studies reporting social support to be a variable that influences nurses' mental health (Hu et al. 2022), making these protective factors increasingly important in both research and clinical practice. On the other hand, a significant negative correlation was found between PMH and risk factors such as anxiety and depression, confirming results from previous studies (Bibi et al. 2020; Boufellous et al. 2023; Çeçen and Vatandaşlar 2021; Lukat et al. 2016).

PMH is an essential part of mental health, as it makes the presence of well‐being visible, and measuring it with this instrument could provide a more complete picture of mental health in populations who are exposed to high levels of stress, such as doctors and nurses. Furthermore, validating this scale in a specific population of healthcare professionals, in such an unusual situation as the COVID‐19 pandemic, was something that could not possibly have been done under normal circumstances. The PMH can be used to determine correlations with various risk and protective factors, which would be a great advantage when implementing programmes addressing these protective factors, the aim being mainly to care for mental health by preventing future disorders and carrying out interventions aimed at specific populations' psychosocial well‐being.

Although for most of the population, the COVID‐19 pandemic was a relatively temporary stressful situation, health professionals such as nurses continue to find themselves in a hospital environment that involves continuous risk to their health, in addition to the responsibility that their work involves and the need to react effectively to emergencies. This means that effective tools are needed to monitor their mental health. Since the PMH is a short scale with good reliability, it can be applied easily and periodically to nurses to identify any potential issues that may arise.

## 6 Limitations

The study does have some limitations. Firstly, the peculiar context at the time of the evaluation due to the COVID‐19 pandemic was almost certainly a determining factor for the response capacity and the large number of participants. It would be advisable to consider similar studies in a ‘post‐pandemic’ context to determine whether there are significant changes and whether these changes are reflected in the results. Secondly, obtaining data through self‐reports presents many drawbacks that would have to be overcome with the support of studies accompanied by other informants (coworkers, family members, patients).

## 7 Conclusions

In conclusion, the PMH scale has the necessary reliability and validity to be used in various psychology settings, such as in health care for nursing professionals. The results of the statistical analysis exhibit significant similarities between the original scale applied to a German population and the translation and adaptation used in this study. The means for each item were similar to those from the original version's samples, the internal consistency of the test was as good as in the original version, and there were no significant differences between the German and nursing samples with respect to the PMH. Nor were any differences found in the psychometric properties of the scale compared to data obtained from applying the scale to the general Spanish population. This indicates that through further studies confirming its reliability, it would be possible to use it more extensively. The fact that this is the first application of this scale in nurses also represents a benefit for further research to be carried out with other healthcare professionals.

## 8 Relevance for Clinical Practice

According to the latest data from the Spanish Ministry of Health (Ministerio de Sanidad 2022), there are 221,406 nursing professionals in Spain, of whom 33,036 belong to primary care teams, 171,963 to hospital care, 3959 to the emergency service, 3390 to specialised training and 9058 to other services, many of whom were exposed to high levels of pressure, work and stress during the COVID‐19 pandemic (Martin‐Rodriguez et al. 2022). The situation may have wreaked havoc on their mental health, so it became essential to have an instrument capable of monitoring the general mental health status of this specific population because of the particular characteristics involved during that period and the importance of their work for society (Mendoza Bernal et al. 2023; Román‐Sánchez et al. 2022).

Finding negative correlations with risk factors, as other studies have done (Bibi et al. 2020; Çeçen and Vatandaşlar 2021; Lukat et al. 2016), also allows us to propose new approaches to the study and treatment of disorders such as depression and anxiety. Because the scale is short, easy to administer and easy to score, it can easily be included in practices, projects and intervention programs with the aim of making the concept increasingly usable and common for the scientific community and the general population. Ultimately, it is not only about the validation of a scale, but also about the importance of understanding that mental health is much more than the absence of disease (WHO 2005)—there is a concept that helps us understand and see beyond mere absence of disorders. Incorporating PMH through this scale will allow us to make a more comprehensive approach to mental health. In addition, it is of utmost importance to consider that, as PMH is a relatively new concept, more research is needed to confirm or refute work to date on the subject. These new studies in different, larger populations could provide more tools, determining whether it is possible to universalise the concept of PMH across different cultures and countries.

## Ethics Statement

The study was approved by the ethics committee of the University of Jaén (Reference: DIC.20/9.TFM). All procedures performed in studies involving human participants followed the ethical guidelines of the Spanish Society of Psychology and the principles of the Declaration of Helsinki and its later amendments or comparable ethical standards. Plus, this article does not contain any studies with animals performed by any of the authors.

## Consent

Informed consent was obtained from all individual participants included in the study.

## Conflicts of Interest

The authors declare no conflicts of interest.

## Data Availability

The data sets generated during and/or analysed during the current study are not publicly available to assure the confidentiality and anonymity of the participants, due to the privacy of the same for further research, but are available from the corresponding author upon reasonable request.

## Appendix 1 Positive Mental Health (PMH) in Spanish

**Table:**

	Items	En desacuerdo	Tiendo a estar en desacuerdo	Tiendo a estar de acuerdo	De acuerdo
		0	1	2	3
1	Suelo estar despreocupado y de buen humour				
2	Disfruto de mi vida				
3	En general, estoy satisfecho con mi vida				
4	En general, tengo confianza en mí mismo				
5	Me las arreglo bien para satisfacer mis necesidades				
6	Me encuentro en buen estado físico y emocional				
7	Siento que estoy realmente bien equipado para afrontar la vida y sus dificultades				
8	Gran parte de lo que hago me produce alegría				
9	Soy un ser humano tranquilo y equilibrado				

*Source:* Adapted from Lukat et al. (2016). Creative Commons Attribution 4.0 International Licence. Adapted with permission.
